# MAPS: machine-assisted phenotype scoring enables rapid functional assessment of genetic variants by high-content microscopy

**DOI:** 10.1186/s12859-021-04117-4

**Published:** 2021-04-20

**Authors:** Jesse T. Chao, Calvin D. Roskelley, Christopher J. R. Loewen

**Affiliations:** Department of Cellular and Physiological Sciences, Life Sciences Institute, University of British Columbia, Vancouver, V6T1Z3 Canada

**Keywords:** High-content screening, High-throughput microscopy, Deep learning, Machine learning, Single-cell phenotyping

## Abstract

**Background:**

Genetic testing is widely used in evaluating a patient’s predisposition to hereditary diseases. In the case of cancer, when a functionally impactful mutation (i.e. genetic variant) is identified in a disease-relevant gene, the patient is at elevated risk of developing a lesion in their lifetime. Unfortunately, as the rate and coverage of genetic testing has accelerated, our ability to assess the functional status of new variants has fallen behind. Therefore, there is an urgent need for more practical, streamlined and cost-effective methods for classifying variants.

**Results:**

To directly address this issue, we designed a new approach that uses alterations in protein subcellular localization as a key indicator of loss of function. Thus, new variants can be rapidly functionalized using high-content microscopy (HCM). To facilitate the analysis of the large amounts of imaging data, we developed a new software toolkit, named MAPS for machine-assisted phenotype scoring, that utilizes deep learning to extract and classify cell-level features. MAPS helps users leverage cloud-based deep learning services that are easy to train and deploy to fit their specific experimental conditions. Model training is code-free and can be done with limited training images. Thus, MAPS allows cell biologists to easily incorporate deep learning into their image analysis pipeline. We demonstrated an effective variant functionalization workflow that integrates HCM and MAPS to assess missense variants of *PTEN*, a tumor suppressor that is frequently mutated in hereditary and somatic cancers.

**Conclusions:**

This paper presents a new way to rapidly assess variant function using cloud deep learning. Since most tumor suppressors have well-defined subcellular localizations, our approach could be widely applied to functionalize variants of uncertain significance and help improve the utility of genetic testing.

## Background

Extrapolating quantitative data from morphological observations enables rigorous statistical analyses. Thus, it is the goal of all cell biology studies. This step needs to be carried out objectively, so that one can interrogate the effects of different experimental conditions or variabilities between samples. Cell biologists typically quantify image data by measuring predetermined criteria such as cell size, cell shape or fluorescence signal intensity. Recent technological advancements in microscopy, including higher resolution and better automation, have empowered us to capture images in superior detail and with greater throughput. However, these advancements also significantly increase the data burden. The conventional workflow of manually adjudicating or measuring cellular and subcellular phenotypes can no longer keep pace with the increasing data load. As a result, demands for automated image analysis solutions have surged.

Computational image analysis techniques, which are a part of the larger interdisciplinary field of computer vision, can be grossly divided into those that utilize machine learning algorithms and those that do not. Classical computer vision processes are stable and efficient, and are already widely used by cell biologists since many of them are pre-packaged into open software platforms like ImageJ and CellProfiler [1]. In comparison, machine learning involves iterative cycles of training and fitting that simulates the human learning process of decision making, making them more flexible at the expense of computational complexity and training time. For instance, given a task of segmenting cells in microscopy images (i.e. detecting individual cell boundaries), classical computer vision techniques include thresholding, edge detection or watershed, while machine learning techniques include clustering, artificial neural network, random forest or support vector machine [2]. All methods can achieve good performance if they are well-suited and fine-tuned for the task. Also, different techniques are frequently used in concert when building up an image analysis pipeline. Choosing the right algorithms therefore requires experience and empirical testing.

Machine learning, in particular, is an attractive method for classifying image-based phenotypes due to its ability to extrapolate patterns in the data and make predictions. This approach has been used to screen cell size mutants and to screen small-molecule therapeutics [3, 4]. The success of machine learning models is dependent on careful feature engineering in which quantitative measures such as cell shape, pixel intensity and texture are derived from single-cell images [5]. However, these features need to be predefined, and the initial high-dimensional feature space will require feature selection and feature reduction before it can be effectively used to train a machine learning classifier [6]. Thus, this type of analysis pipeline is usually hand-tuned for each dataset and cannot easily incorporate new data or be transferred to an entirely new dataset.

To overcome this challenge, a specialized branch of machine learning, deep learning, has recently gained momentum in the computer vision field. Deep learning is based on multiple layers of artificial neural networks and does not require features to be predefined. Instead, a series of convolutional filters are incorporated into the network (i.e. convolutional neural network (CNN)) to extract pixel-level features for training and classification [7]. This learning structure is inherently flexible at handling a wide variety of image data. Trained networks can also be updated with new data through transfer learning [8]. Thus, CNNs have accelerated computer vision research because of their ability to solve challenging biomedical image analysis problems, such as 2D/ 3D cell segmentation, organelle segmentation, anatomical segmentation, cell detection, false fluorescent labeling or feature extraction [9–13]. For these reasons, deep learning techniques are well-suited for automating the analysis of high-content microscopy (HCM) data, in which high information content is captured for each sample [14]; or high-throughput microscopy data, in which multiple samples are imaged in rapid succession [15]. For example, CNNs was used to classify the localizations of fluorescently-tagged proteins in yeast from HCM images with superior accuracy [10, 14].

Nevertheless, applying deep learning requires substantial computational expertise. We were motivated to lower this technical threshold. Therefore, in this manuscript, we present an image analysis pipeline that uses cloud deep learning tools that require very little programming. We named the pipeline MAPS for machine-assisted phenotype scoring, and applied it to score changes in protein localizations caused by genetic variations from HCM data.

Many different strategies have been undertaken to classify genetic variants. The gene-specific approach is to develop an assay that interrogates the biochemical function of the protein product, followed by quantitatively measuring such function in variants. This strategy has been applied to *BRCA1* variants, where the homology-directed DNA repair function of BRCA1 is the key measure [16]; to *EGFR* variants, where the transforming potential of EGFR is used to assess its mutants [17]; and to *TP53*, where the anti-proliferative function of p53 is used to annotate its variants [18]. In contrast, the gene-agnostic approach is to develop a generalizable assay that exploit the universal attributes of gene products. One technique, called VAMP-seq, measures the relative intracellular abundance of the expressed protein, where lower expression is indicative of loss of function caused by the genetic variation [19]. Gene expression profiling has also been used to fingerprint the molecular functions of a gene and to reveal changes induced by its variants [20].

Previously, we developed a gene-specific assay for the tumor suppressor *PTEN* [21]. While the assay is clinically relevant and scalable, we wanted to engineer a generalizable assay that can be used to functionally assess potentially any gene without needing prior knowledge of gene function. It is well-recognized that the subcellular localizations of proteins are usually crucial for their functions. For instance, the DNA repair activities of p53 and BRCA1 are dependent on their localizations to the nucleus and mutations that disrupt their localizations will significantly impede their functions [22]. Thus, we hypothesized that screening for mutations that alter a protein’s wildtype localization could potentially help discover evidence of pathogenicity. Based on this principal, we assed PTEN variants using automated widefield fluorescent microscopy. We then demonstrated using MAPS to rapidly classify variant phenotypes from the large amount of microscopy data. Our new method for assessing variants is simple, scalable and effective, and our software can help the research community more easily utilize deep learning to automate image analysis.

## Results

### Variant assessment workflow

**Fig. 1:**
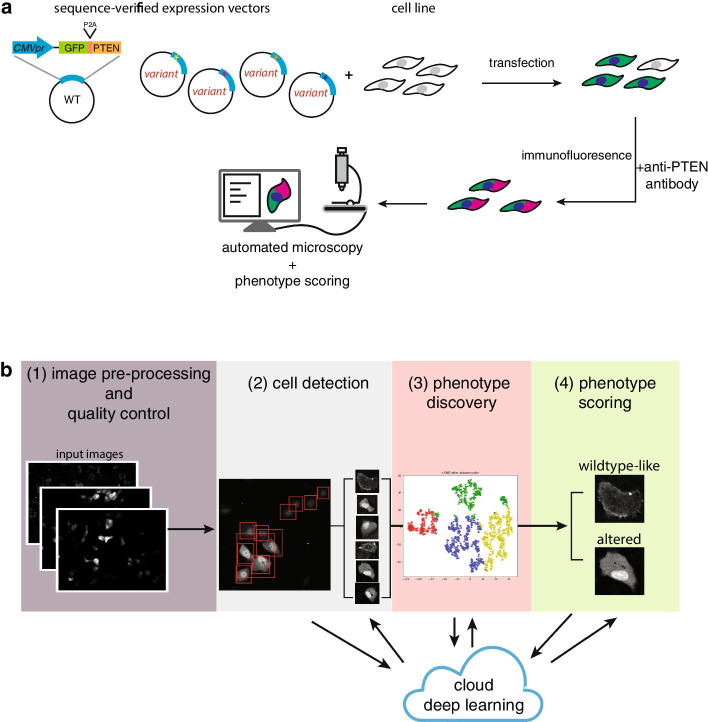
Overview of the experimental and analytical setup. **a** Workflow for expressing and visualizing variants via IF and automated widefield fluorescent microscopy. **b** MAPS software contains the following four modules: (1) image quality control (see Fig. 3); (2) cell detection (see Fig. 4); (3) phenotype discovery (see Figs. 5, 6); and (4) phenotype scoring (see Fig. 7). All figures are generated using Adobe Illustrator version 23.0.6

We first established the workflow for visualizing the localizations of *PTEN* variants (Fig. 1a). We cloned different *PTEN* alleles into an expression vector that expresses GFP and PTEN as a fusion protein interspersed by a P2A self-cleaving peptide, the same design as we previously published [21]. GFP and PTEN were then expressed as individually folded proteins in 1:1 ratio. After transfection, we carried out immunofluorescence (IF) to visualize PTEN localizations. Finally, we used automated widefield fluorescent microscopy to capture images and used MAPS to perform automated image analysis and phenotype scoring.

MAPS includes the following modules: (1) image quality control, (2) cell detection, (3) feature extraction/phenotype discovery and (4) phenotype scoring (Fig. 1b). Each of these modules can be executed independently, allowing users to incorporate or substitute their preferred software such as CellProfiler, ImageJ or MATLAB scripts into the pipeline. We organized each MAPS module as a standalone Jupyter Notebook, which provides an interactive interface for fine-tuning parameters. This is similar to the design of the Allen Cell Structure Segmenter [23]. To give a brief overview, the first deep learning model will perform cell detection and isolate regions of interest (ROIs). Next, the second deep learning model extracts features from each ROI, and ROIs with similar features are clustered to help the user discover and define novel phenotypes. Finally, a third deep learning model will classify all cells identified by the cell detection module.

### Loss of function mutations alter the subcellular localization of PTEN

**Fig. 2:**
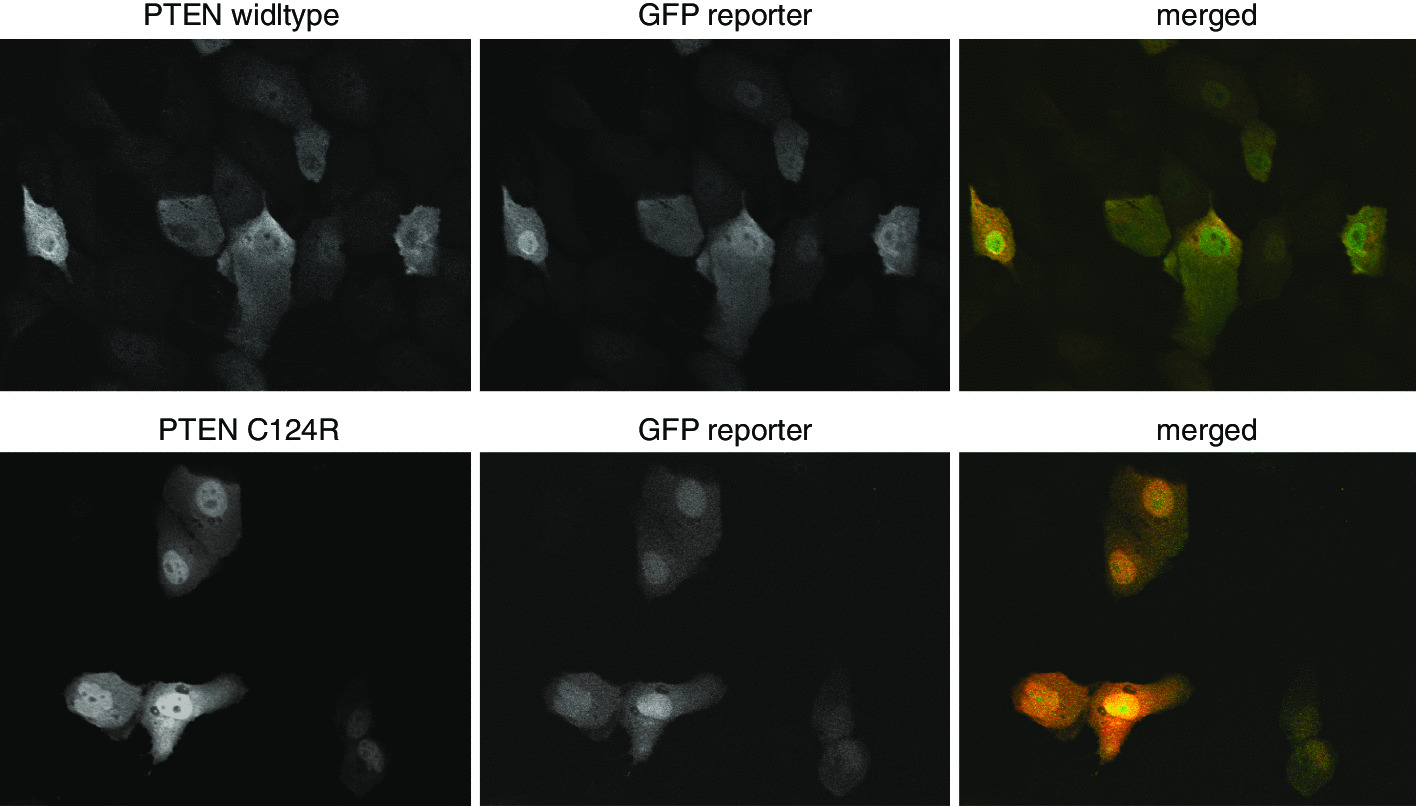
Representative microscopy images showing the localizations of wildtype and C124R allelic variant of PTEN. PTEN and the GFP reporter were expressed in 1:1 ratio. PTEN was visualized via IF

We carried out pilot experiments to test the pipeline. We first localized PTEN in the non-tumorigenic human breast epithelia cell line MCF10A via IF. Wildtype PTEN has been reported to shuttle between the cytoplasm and the plasma membrane (PM), which is essential for its tumor suppressor function in dephosphorylating phosphatidylinositol-3,4,5 trisphosphate (PI(3,4,5)P_3_) [24]. Although PTEN does not contain a canonical nuclear localization signals (NLS), nuclear PTEN is apparent in quiescent cells, but not typically found in dividing cells [25]. Consistently, we noticed that wildtype PTEN localized mostly in the cytosol with minor PM staining, but is excluded from the nucleus. In contrast, the localization of a known tumour-associated non-functional variant, C124R [26, 27], was predominantly nuclear (Fig. 2). Since there were clear differences in the localizations of selected PTEN variants, we felt confident to use alterations in PTEN localizations as the key phenotypic measure for scoring variant function.

### Module 1: Image quality control

**Fig. 3:**
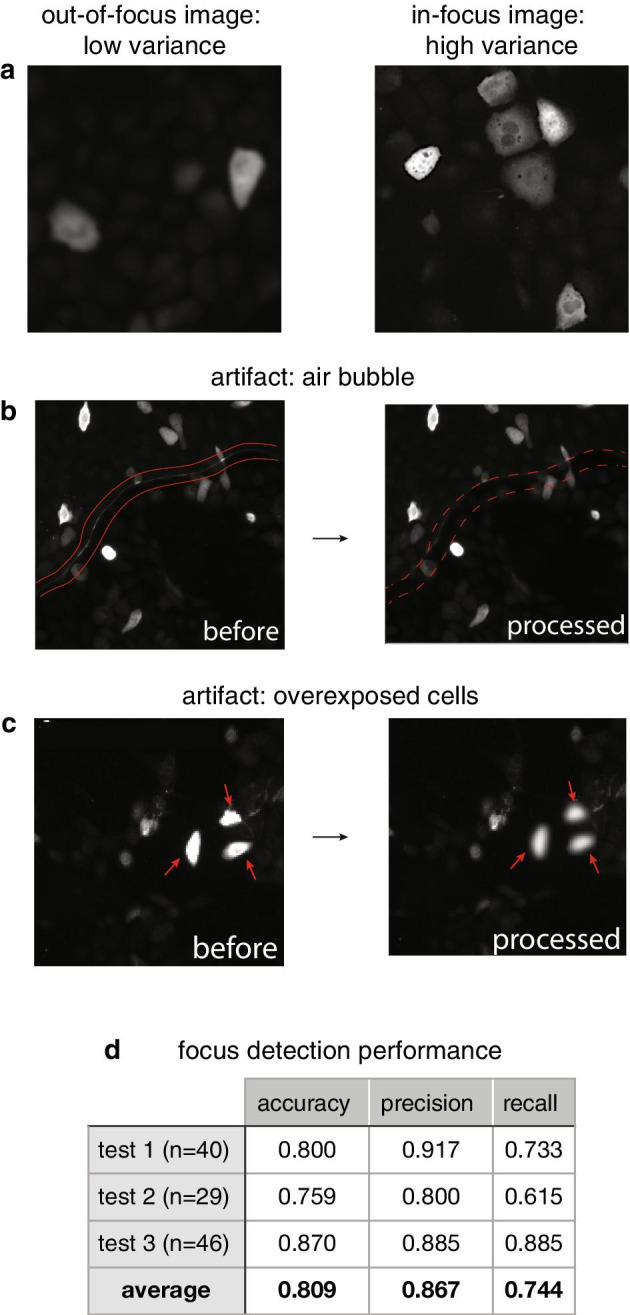
Module 1: image quality control. **a** Laplacian variance was used as a focus measure operator to differentiate blurry and in-focus images. **b**, **c** Aberrations such as air bubbles or overexposed cells interfered with focus measure calculations and were further processed to obtain accurate focus measures. **d** Performance metrics from three independent tests. Test images were randomly sampled from all experiments

Next, we acquired images using automated microscopy. We expected that automated microscopy instruments will occasionally not focus on the desired focal plane. Also, they do not discriminate against images containing aberrations such as air bubbles, scratches or foreign fibers. To ensure the quality of downstream analyses, we implemented quality control (QC) measures to remove low quality images. Performing image QC by visual inspection is difficult because automated microscopy generates gigabytes of image data. Thus, manually screening HCM data is time consuming and undesirable. A number of strategies are commonly used to perform image QC, such as building custom software solutions [28] or implementing a QC pipeline in CellProfiler’s MeasureImageQuality module [29]. In order to integrate seamlessly with the other modules, we decided to build custom functions to compute focus measures using variance of Laplacian to calculate the amount of edges in an image. In-focus images will generate high variance, while blurry images will have low variance (Fig. 3a) [30, 31]. However, air bubbles or overexposed cells (due to cells overexpressing the target protein) will have well-defined edges which interfere with focus measure calculations. To overcome these issues, we implemented image dilation to remove the edges from air bubbles (Fig. 3b), and masking followed by Gaussian blurring to remove edges from overexposed cells (Fig. 3c). We performed three separate focus detection tests to challenge our QC module in removing blurry images. The true negatives were blurry images that were correctly removed, and false negatives were in-focus images that were incorrectly removed. The true positives were in-focus images that were not removed, and false positives were blurry images that were not removed. On average, our QC module can remove blurry images with an accuracy of 0.809, precision of 0.867 and recall of 0.744 (Fig. 3d).

### Module 2: Cell detection using cloud deep learning

**Fig. 4:**
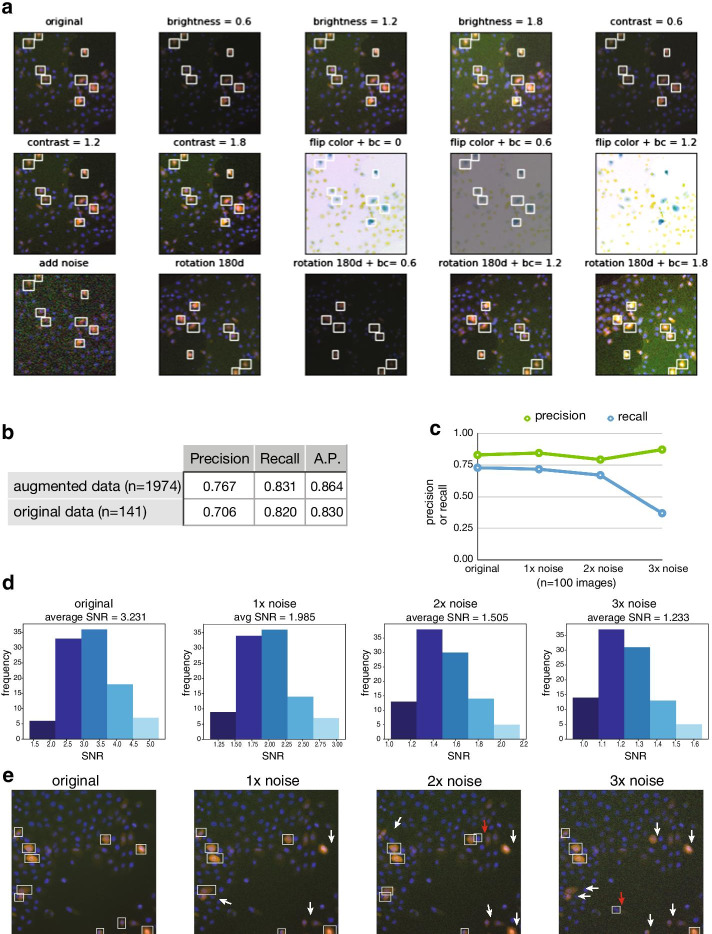
Module 2: cell detection and training augmentation. **a** Sample images from training augmentation. The original image (top left) underwent 14 different transformations including brightness and contrast (bc) adjustments, color inversion (flip color), noise addition and image rotation. **b** The precision, recall and average precision (A.P.) of our cell detection model after and before training augmentation. **c** The precision and recall metrics of our model at detecting 100 test images at increasing noise levels. **d** The frequency distributions of the SNR of the 100 test images at increasing noise levels. **e** A test sample illustrating the model’s ability to detect cells at increasing noise levels. White arrows, relevant ROIs that were not detected at higher noise. Red arrows, irrelevant ROIs detected

After image QC, we needed to isolate individual cells as ROIs. The goal is to later group ROIs with similar PTEN localization patterns together in Module 3 so that we can define distinct phenotype classes. These classes will then be used to train the final classification model in Module 4. Cell detection places bounding boxes around each ROI and is different from cell segmentation which aims to identify cell boundaries as defined by the plasma membrane (e.g., in mammalian cells) or by the cell wall (e.g., in yeast cells). Although in certain cases cell segmentation would be desirable, such as when the cell culture is confluent, cell detection was well-suited for our application since we used sub-confluent cultures. To carry out cell detection, we took advantage of the Custom Vision module of Azure, Microsoft’s cloud-based machine learning platform. We trained a cell detection model on Azure and used the endpoint to predict bounding boxes. This set of training images (n = 141) were obtained from the wildtype *PTEN* localization experiment, and the ground truth labels were 530 manually labeled bounding box coordinates. Bounding boxes were drawn using the Azure Custom Vision graphical user interface. Both the training data and the .csv file containing the ground truth bounding box coordinates are available in our GitHub repository. Training took ~ 30 min on Azure, and this preliminary model showed reasonable performance with precision = 0.706, recall = 0.82, and average precision (A.P.) = 0.83. A.P. is the area under the precision-recall curve [32]. To improve the performance of this preliminary model, we implemented data augmentation, a common technique used in deep learning to boost the training data [9]. We implemented 14 different image transformations techniques including image rotation, flipping, contrast adjustments, color inversions and adding noise (Fig. 4a), and boosted the original training data to 1,974 images. Training on the augmented dataset took ~ 45 min and raised the precision to 0.767, recall to 0.831 and A.P. to 0.864 (Fig. 4b).

We next interrogated the noise threshold of the Azure object detection model. We assembled a test set with 100 images, and increased the image noise step-wise which lowered the signal to noise ratios (SNR) of the test set (Fig. 4c, d). We found that although the model maintained precision, recall gradually decreased at higher noise levels to eventually failing catastrophically at 3 × noise (Fig. 4c) or average SNR below 1.5 (Fig. 4d). Using a sample image from the test set as an example, we saw that as the SNR decreased the model was still able to detect PTEN-expressing cells (Fig. 4e, white boxes) with precision and only detected one irrelevant ROI at 2 × and 3 × noise (Fig. 4e, red arrows). However, as the noise level increased the model failed to detect all relevant ROIs (Fig. 4e, white arrows), suggesting lower recall or sensitivity. Thus, from our test results we would suggest keeping the average SNR of input images above 1.5 for best performance.

### Module 3: Phenotype discovery

Earlier, we observed that wildtype PTEN was nuclear-excluded, while the non-functional C124R variant was nuclear-enriched (Fig. 2). Both of these localization patterns have been previously reported [24]. However, we did not know whether other variants might induce additional PTEN localization. Thus, in Module 3 we will explore novel phenotypes. The objectives of phenotype discovery are to: (1) remove noise or outliers; (2) inspect the detected ROIs for novel phenotypes in order to (3) define the class labels and formulate the training data for the final phenotype classification model in Module 4.

**Fig. 5:**
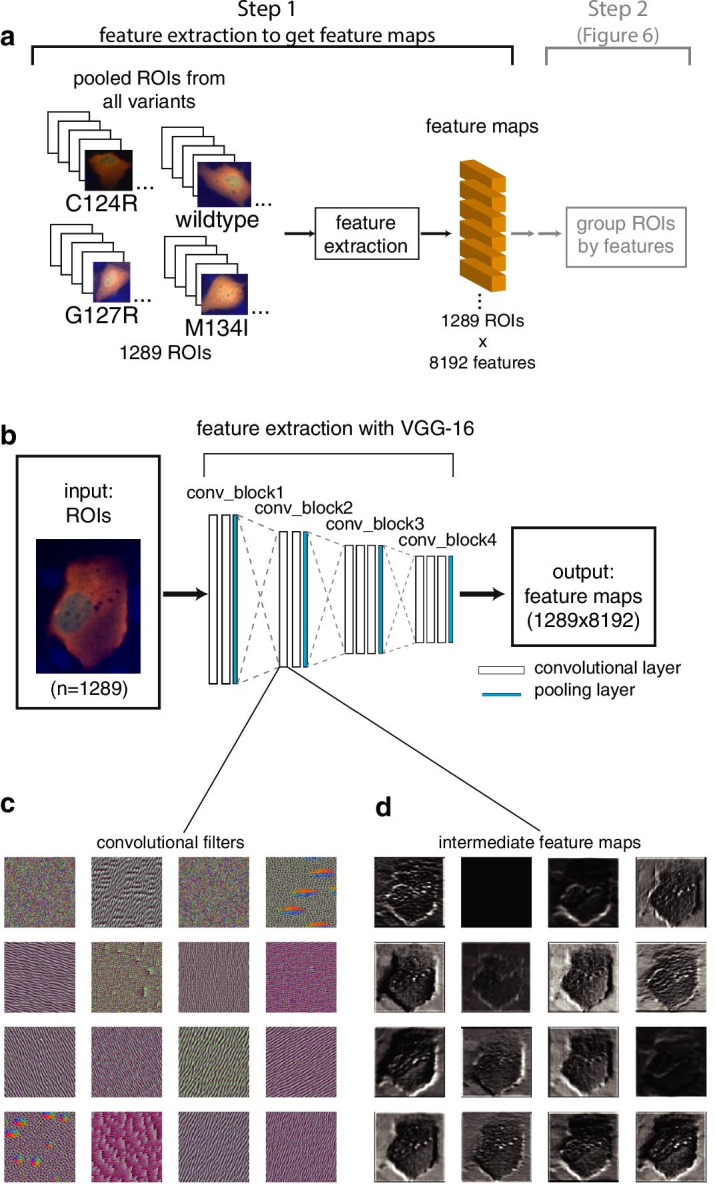
Module 3: phenotype discovery. **a** Overview of this module. In step 1, ROIs from all variants were pooled and their feature maps extracted. In step 2 (see Fig. 6), clustering algorithm grouped ROIs with similar features together. **b** Details of the feature extraction process. Each ROIs was processed through a reconfigured and pretrained VGG-16, resulting in a feature map containing 8192 features. **c** 16 of the 128 convolutional filters from the first layer of the second convolutional block of VGG-16. **d** 16 intermediate feature maps extracted by the convolutional filters in **c**

**Fig. 6:**
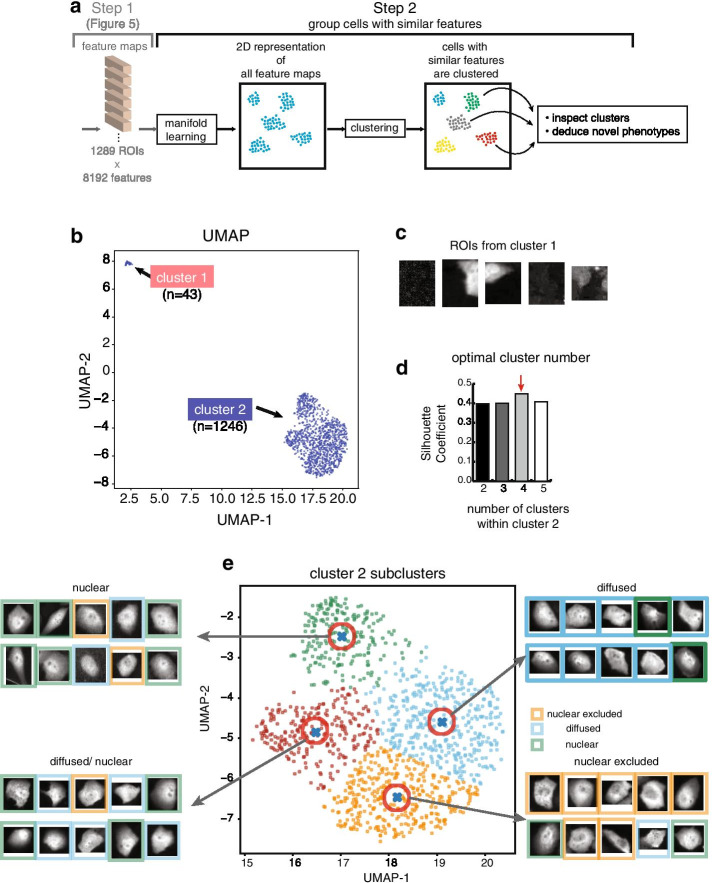
Module 3 continued. **a** Continued overview of this module. After feature extraction (step1, Fig. 5), the dimensionality of the feature maps was reduced, and data points were embedded in a 2D manifold for visualization. Then, cells with similar features were grouped together by clustering. **b** A 2D embedding by UMAP showing two clusters of data. **c** ROIs from the smaller cluster were noise and were discarded. **d** Mean Silhouette Coefficient for all datapoints in the larger cluster, cluster 2, were computed at different cluster numbers. The highest score indicated the optimal cluster number. **e** Cluster 2 was further partitioned using spectral clustering into four subclusters. For each subcluster, 20 nearest neighbors of the centroid (blue crosses) were visually inspected and their PTEN localizations were manually classified. 10 were plotted, and the predominant PTEN localization for that cluster was indicated

Operationally, the phenotype discovery process consists of two steps. In step 1, we extract features from each ROI using convolutional filters, shown in Fig. 5a; in step 2, we group ROIs with similar features together using unsupervised machine learning techniques, shown in Fig. 6a. To begin step 1, we first pooled ROIs from different variants together and randomly sampled 1,289 ROIs. Per ROI feature maps were extracted through a reconfigured VGG-16 model, a very deep convolutional neural network [33]. Since we only needed VGG-16 for feature extraction and not image classification, we reconfigured VGG-16 to use its first four convolutional blocks with weights from pretraining with ImageNet [34] (Fig. 5b). To illustrate the feature extraction process, we visualized 16 convolutional filters from the first convolutional layer of the second convolutional block of VGG-16 (Fig. 5c). We also plotted the intermediate feature maps extracted from the 16 convolutions (Fig. 5d).

The extracted feature maps (1289 ROIs × 8,192 features) were of very high dimensions. To begin step 2, we reduced the dimensions from 8192 to 30 using UMAP, a manifold learning technique [35]. Dimensionality reduction with UMAP enabled efficient clustering. As a result, a 2D manifold of the reduced feature maps showed two distinct clusters (Fig. 6b). Upon inspection, the ROIs in the smaller cluster (n = 43) were noise and should be eliminated (Fig. 6c), while the larger cluster (n = 1246) contained useful ROIs and required further partitioning. To determine the ideal number of sub-clusters, we calculated the mean Silhouette Coefficient [36]. The highest Silhouette Coefficient was achieved at 4 sub-clusters, indicating that the tightness and separation of all data was optimal (Fig. 6d). Thus, we applied spectral clustering [37] to partition the larger cluster into 4 sub-clusters (Fig. 6e).

We next visualized representative ROIs from each sub-cluster. We inspected 20 nearest neighbors of each of the centroids (Fig. 6e, blue crosses), and found that they shared similar PTEN localization patterns within the cluster. The green cluster (1) showed cells with predominantly nuclear-localized PTEN; the blue cluster (2) showed cells with diffused PTEN localizations; the orange cluster (3) showed mostly nuclear-excluded PTEN; the dark red cluster (4) showed a mixture of diffused and nuclear PTEN (Fig. 6e). All in all, we discovered three major PTEN localization patterns amongst the ROIs from all tested variants: nuclear, nuclear-excluded and diffused. We defined these three class labels as the ground truth dataset for training the phenotype classification model in Module 4.

### Module 4: Phenotype classification using cloud deep learning

**Fig. 7:**
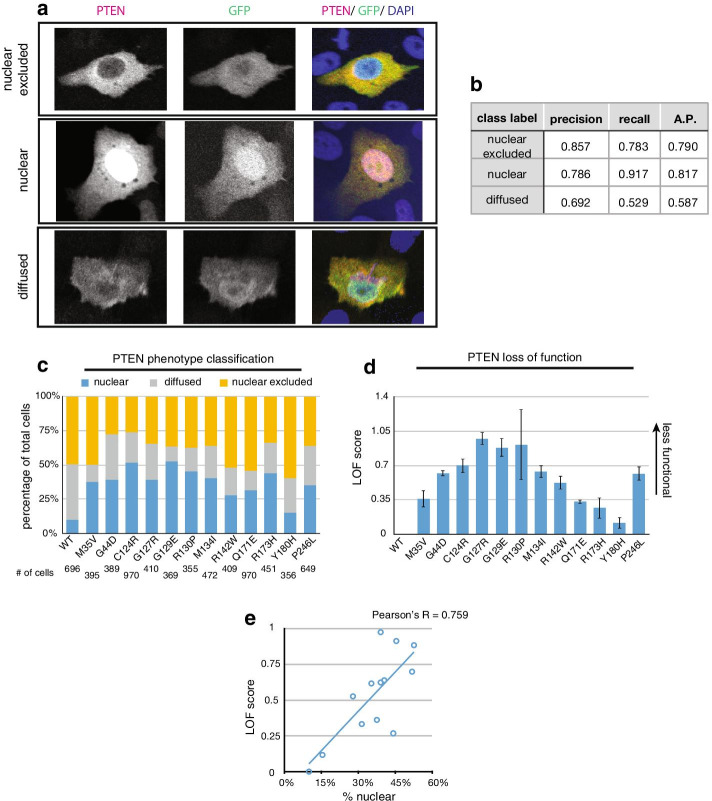
Module 4: phenotype scoring identified loss of function PTEN variants. **a** Representative ROIs of the three PTEN localization classes that were used to train a classification model on Azure. **b** The performance metrics of the phenotype classification model for each class label. **c** Results of automated phenotype scoring for wildtype PTEN and 12 variants. **d** LOF scores for the same variants as in **b** taken from a previously published spheroid assay [21]. **e** Scatter plot showing the correlations between the LOF scores and the percentage of cells with nuclear PTEN. The best fit line is also shown

Using the three phenotype classes defined in Module 3, we curated a training dataset consisting of > 100 ROIs per class and their labels as ground truth (Fig. 7a). Training an image classification model on Azure with this dataset took ~ 45 min. The performance for this model was listed in Fig. 7b. Next, we used the trained model to perform automated phenotype scoring on wildtype PTEN and 12 variants: M35V, G44D, C124R, G127R, G129E, R130P, M134I, R142W, Q171E, R173H, Y180H and P246L (Fig. 7c). We noticed that a low percentage of wildtype cells showed nuclear PTEN localization (~ 10%); in contrast, known pathogenic variants including C124R, G127R, G129E and R130P had much higher nuclear PTEN (> 50%, pathogenicity classifications taken from ClinVar). This was consistent with our initial observation (compare Figs. 2, 7c). We hypothesized that nuclear PTEN accumulation could be indicative of loss of function (LOF). Thus, we compared the localization distributions to the LOF scores that we previously measured for these variants using a spheroid assay (Fig. 7d [21]). Notably, the percentage of cells with nuclear PTEN correlated strongly with LOF scores (Fig. 7e, Pearson’s correlation = 0.759, *p* = 0.003). There were two variants that stood out. First, the variant R173H had a low LOF score but high nuclear PTEN. This suggested that the R173H mutation did not sufficiently disrupt PTEN’s physiological function in the context of anchorage independent cell adhesion, but it did alter PTEN’s subcellular localization. Second, the G127R variant had the highest LOF score, but its nuclear PTEN was no higher than that of G44D or M134I. Considering that G127R is classified as likely pathogenic according to ClinVar and so is M134I (G44D is pathogenic), the two assessment methods both indicate that G127R as LOF although they produce scores of different magnitudes. Hence, we reasoned that assessing variant function by subcellular localization could complement the spheroid assay to increase the overall detection sensitivity. In conclusion, we reasoned that the gene-agnostic localization scoring method could be an effective replacement of the gene-specific spheroid assay.

## Discussion

The current strategies for assessing variant functions, including in silico predictions or in vitro testing, all have limitations. In silico prediction software are computational classifiers that utilize calculated features such as amino acid properties [38], protein sequence conservation [39], protein stability [40], or evolutionary genomic features [41] as inputs. Certain software, like Polyphen-2, combine multiple streams of information to improve performance [42]. This type of software is capable of processing a large number of sequence variations very quickly. Although versatile, their generalist designs and theoretical assumptions result in different models frequently producing conflicting classifications [43]. Studies using independent datasets to benchmark various in silico models indicate that their real-world performances are likely lower than originally reported [44, 45]. In comparison, in vitro testing methods consume more time and resources. They are also regarded to be more reliable [46], as they utilize gene-specific assays to directly interrogate the effects of sequence variations on protein function. Although each assay is tailored to the physiological function of the gene, this approach requires significant investments in development and execution. We also chose the in vitro approach when we previously developed a 3D tumor spheroid assay for functionalizing *PTEN* variants. Hence, we sought to merge the merits of both approaches when developing MAPS by scoring alterations in protein localization as a measure of loss-of-function.

The most prominent function of PTEN is its ability to dephosphorylate PI(3,4,5)P_3_ which is the main product of the PI3K/ AKT pathway that promotes cell survival and proliferation [47]. Therefore, loss of PTEN’s lipid phosphatase activity will have significant consequences in tumor initiation and progression. Since the main intracellular pool of PI(3,4,5)P_3_ is on the PM [48], we can expect to find PTEN localizing to the intracellular side of PM. Consistently, PTEN has been found to traffic between the cytoplasm and PM [24]. Correct PTEN subcellular localization is therefore crucial for its anti-malignancy effects. However, different studies have documented conflicting sightings of PTEN localizations. On one hand, nuclear accumulation of PTEN has been detected in invasive breast tumors [49], and certain cancer-derived PTEN missense mutations result in an increase of nuclear PTEN [50]. On the other hand, the absence of nuclear PTEN has also been reported in other types of solid cancers as well as cell lines [51, 52], adding to the confusion of whether nuclear PTEN is associated with tumorigenicity. It was later determined that wildtype PTEN containing no mutations should be predominantly nuclear in quiescent cells such as neurons or those in G_0_-G_1_, but mainly cytoplasmic in actively dividing cells such as tumor cells or those in S phase [25]. Since our assay used sub-confluent cultures of non-tumorigenic MCF10A cells, our observation that wildtype PTEN was mostly nuclear-excluded was consistent with the literature. Therefore, we reasoned that our assay design was well-suited for assessing PTEN function based on disruption of its cytoplasmic localization. Our approach is then an excellent platform that enables the systematic survey of the subcellular localization of PTEN allelic variants. By automating phenotype scoring, we alleviated the constraint in data processing and analysis. This allowed us to focus on generating a variant library which is the bottleneck of all variant assessment workflow.

Our phenotype scoring pipeline has a number of differences than the convention. A common strategy for scoring phenotypes involves first quantitatively measuring predefined cell-level morphological features. Then, the features are handed over to a classical ML algorithm such as random forest or SVM for classification [5, 53]. Since the features, such as cell size, texture, shape or protein fluorescence intensity [5] are manually defined, the classification may be unsuccessful or biased if important features were missed. This approach was the standard practice before deep learning became mainstream. In contrast, our deep learning approach involves using learned convolutional filters to extract relevant features to perform classification [12]. This is a more flexible solution because the same CNN architecture can be trained on different datasets to perform distinct image classification tasks. Importantly, CNNs do not require an expert to pre-select features. However, hardware and software limitations often prevent the wide-spread adoption of deep learning in image analysis pipelines. On the hardware side, recent deep learning libraries benefit significantly from having GPU (graphical processing unit) acceleration during model training, but GPU hardware is expensive. On the software side, programming CNNs is not trivial even with the release of high-level deep learning libraries such as Keras and PyTorch. In the past, these considerations have driven scientist to develop generalist CNN models that have been trained to detect all possible subcellular protein localizations, so that the community can utilize the trained end point to classify the localization of their protein of interest. Some examples of this approach are DeepLoc, a CNN model that can classify 15 localizations in budding yeast [14], or DeepYeast, which can classify 12 localizations [10]. Nevertheless, these pretrained networks require transfer learning [8] to work on images obtained from different microscopy instruments. They also may not perform when the cell types are different. Therefore, an easily retrainable model without stringent hardware requirements will mitigate all of these issues.

Our goal was to make MAPS adaptable and applicable. Building deep learning models should be simple and intuitive. Therefore, we adopted cloud deep learning to eliminate hardware and software barriers. As a result, MAPS can help different user easily train and deploy a new experiment-specific model. Since imaging experimental conditions vary greatly and can involve a variety of cell types, fluorescent labels and instruments, it is more practical to quickly build specific models rather than using pretrained ones. Cloud platforms also has the advantage of providing consistent training and prediction performances at a fraction of the cost of purchasing and maintaining GPU-capable local machines. We tested the three leading cloud machine learning platforms including Microsoft Azure, Amazon Web Service (AWS) and IBM Watson, and found that Azure has the most intuitive graphical user interface. Importantly, the Custom Vision module on Azure allows users to create object detection or classification models entirely code free, and can achieve reasonable performance using very few (~ 20) training images. This is immensely more convenient than building deep learning models from scratch which typically requires hundreds if not thousands of manually labeled images for training and validation [7]. Thus, we chose to implement Modules 2 and 4 on Azure. Module 3, phenotype discovery, was implemented using the prebuilt and pretrained VGG-16 model from Keras and runs on Google’s Colab GPU which is currently free. Therefore, MAPS can serve as the foundation for building a custom deep learning image analysis pipeline at low cost.

So far, functional characterizations of variants have relied on specific assays tailored to interrogate each protein’s function. For instance, as homology-directed DNA repair is crucial for BRCA1′s tumor-suppressing activity, it is often used to assess the functional consequences of BRCA1 variants [16, 54]. Nonetheless, the DNA repair activities of BRCA1 are also dependent on its import into the nucleus via two nuclear localization signals (NLS) [55], and cancer-associated mutations often disrupt its nuclear import [56]. Similarly, p53 has 3 NLS and its import into the nucleus is also required for its function in suppressing malignant transformation [57]. In fact, most tumor suppressors have well-defined subcellular localizations that become altered in cancer (see [58] for summary). It is important to note that loss of protein localization is used clinically to facilitate cancer diagnosis and prognosis. For example, cell membrane-associated tumor suppressors such as Cadherin-1 and beta-catenin play important roles in maintaining cell adhesion, and loss of their PM localization and nuclear accumulation is present in a wide variety of solid tumors and is associated with poor prognosis [59–62]. Additionally, a class of cancer therapeutics specifically targets protein localization as its mechanism of action. For example, selective inhibitors of nuclear exporters are small molecules that increase the nuclear retention of p53 and p21 [63]. Thus, we anticipate our approach of detecting loss-of-function variants by screening variant localizations can be broadly applied to other tumor suppressors.

All in all, we not only developed a new framework for rapidly assessing the functional effects of genetic variations at scale, but also provided an accessible way for cell biologists to automate image analysis with deep learning. Our code base can be immediately useful to the research community to leverage the intuitive creation and flexible deployment of deep learning models on the Azure cloud platform. We think our work will help biologists expand their capacity of handling the increasing amount of image data and will help drive the throughput of more complex microscopy-based studies.

## Conclusions

We developed MAPS to automate phenotype scoring of HCM data and used it to identify loss-of-function genetic variants. MAPS stands out for other software tools by helping users build custom deep learning models using Microsoft’s Azure cloud computing platform, completely code-free. Also, the computation-intensive steps are carried out by cloud GPUs which significantly accelerate computation and lowers the hardware requirements of the user’s local machines. We think MAPS can help empower cell biologists with the analytical power of deep learning. Finally, assessing variant function using microscopy is a simple and easily scalable approach, and is a more cost-effective alternative than developing gene-specific assays.

## Materials and methods

For detailed instructions on our immunofluorescence workflow, please see: https://dx.doi.org/10.17504/protocols.io.bn68mhhw

### Cell culture

The *PTEN*–/– cell line (MCF10A background) was purchased from Horizon Discovery and verified by western blotting. Cell were cultured according to published protocols [64] and were maintained in a 37 °C incubator with 5% CO2. Mycoplasma was tested monthly by direct DNA staining with DAPI.

### Plasmids and transfections

PTEN expression vectors were generated as previously described [21]. Transfection was carried out 24 h after seeding 50,000 cells in a 12-well dish containing 22 × 22 mm glass coverslips (Thermo Fisher Scientific) using Lipofectamine 2000 (Thermo Fisher Scientific) according to manufacturer’s protocols. Successful transfection was confirmed by direct visualization of GFP expression using a fluorescent microscope.

### Immunofluorescence

24 h after transfection, cells were fixed using 4% paraformaldehyde in PBS. Cells were permeabilized with 0.1% triton x-100 in PBS, blocked with 10% BSA, and incubated overnight with rabbit anti-PTEN antibody (138G6, Cell Signaling Technology). Coverslips were then incubated with mouse anti-rabbit Alexa Fluor 568-conjugated antibody (Invitrogen), followed by DAPI, and mounted using ProLong Gold antifade mountant (Thermo Fisher Scientific).

### High-content microscopy

Images were acquired using a Cellomics Arrayscan (Cellomics Inc.). using a 20 × objective. A minimum of 500 images were acquired per coverslip at 3 channels (green/ red/ blue) per image.

### Notes on algorithms

#### Deep learning models

For image recognition tasks, deep CNNs are usually the de facto choice in modern pipelines for their proven performance and efficiency. We chose Microsoft’s Azure Custom Vision to perform cell detection (Module 2) and phenotype classification (Module 4) because they provide a user-friendly graphical interface for model training and validation. The process is completely code-free. Additionally, users can access a cloud GPU instance at a reasonable cost which significantly accelerates the workflow. For these reasons, Azure is a sensible choice that will appeal to a wide audience in the cell biology field. For Module 3, we opted to use VGG-16, a well-recognized deep CNN architecture with over 52,000 citations (at the time of this writing), to perform feature extraction [33]. 1.1.1. *Noise operations.*

To add noise to images, we used NumPy’s random sampling routine to add Gaussian noise. We adopted the common convention for estimating SNR in image processing [65]:

$${\text{SNR = }}\frac{{{\text{Mean}}\,{\text{pixel}}\,{\text{value}}}}{{{\text{Standard}}\,{\text{deviation}}\,{\text{of}}\,{\text{pixel}}\,{\text{values}}}}.$$

#### VGG-16 modifications

We removed the last convolutional block and the fully connected layers from VGG-16 because we did not need to perform classification with VGG-16. Model weights were pre-trained on ImageNet [7]. Each ROI is scaled up to 148 × 148 during pre-processing.

#### UMAP hyperparameters

To preserve global data structures, we followed the UMAP documentations and set n_neighbors = 100 and min_dist = 0.1. We used the Chebyshev distance metric. We also used PCA initialization to reduce feature dimensions down to 500 before UMAP.

### Computational requirements

MAPS was written in Python 3.6.10. Other libraries include NumPy (1.18.1), pandas (1.0.3), opencv-python (4.1.1.26), matplotlib (3.1.3), UMAP (0.5.0), and Keras with TensorFlow backend (2.4.1). Azure is accessed using Microsoft Azure Custom Vision SDK (3.1.0). For detailed instructions on using MAPS, please see our Jupyter notebooks at https://github.com/jessecanada/MAPS/. For detailed implementation guide, please visit our protocols.io article at https://dx.doi.org/10.17504/protocols.io.bn7dmhi6.

## Data Availability

The software and datasets generated during and/or analysed during the current study are available in our GitHub repository, https://github.com/jessecanada/MAPS/.

## Abbreviations

MAPS: Machine-assisted phenotype scoring
CNN: Convolutional neural network
CPU: Central processing unit
DL: Deep learning
GPU: Graphics processing unit
HCM: High-content microscopy
IF: Immunofluorescence
LOF: Loss of function
ML: Machine learning
NLS: Nuclear localization signal
PTEN: Phosphatase and tensin homolog
PM: Plasma membrane
QC: Quality control
ROI: Region of interest
SVM: Support vector machine
UMAP: Uniform manifold approximation and projection
